# Key factors for increased tip-apex distance when treating intertrochanteric fractures with InterTAN nails

**DOI:** 10.3389/fbioe.2024.1426307

**Published:** 2024-11-01

**Authors:** Naifeng Zhu, Lianxia Wu, Xiaofeng Han, Zhonglai Qian

**Affiliations:** ^1^ Department of Orthopaedics, The First Affiliated Hospital of Soochow University, Suzhou, China; ^2^ Department of Orthopaedics, Renji Hospital, School of Medicine, Shanghai Jiao Tong University, Shanghai, China; ^3^ School of Social Development, Population Research Institute, East China Normal University, Shanghai, China

**Keywords:** tip-apex distance, cut-out, implant failure, InterTAN, intertrochanteric fracture

## Abstract

**Background:**

The tip-apex distance is a key factor in predicting implant cut-out after intramedullary fixation for intertrochanteric fractures. This study aimed to evaluate the factors associated with an increased tip-apex distance when treating intertrochanteric fractures using an InterTAN nail.

**Methods and Material:**

We retrospectively analyzed patients with intertrochanteric fractures who underwent InterTAN nail insertion between January 2017 and March 2022 at our hospital. Medical and radiological data were collected. Measurements of preoperative factors and postoperative factors were performed accordingly. Multivariate logistic regression analysis was performed to determine the statistically significant variables of the tip-apex distance.

**Results:**

This study included 102 patients with intertrochanteric fractures. The average tip-apex distance measured 22.4 ± 7.1 mm, ranging from 9.3 to 48.0 mm. The length of the femoral neck on the non-fractured side, lag screw placement in the sagittal plane (center-inferior, superior) and coronal plane (posterior), and the angle between the line of the proximal nail axis and the femoral long axis were identified to be statistically significant factors for the tip-apex distance.

**Conclusion:**

To obtain a shorter tip-apex distance, we recommend a medial trochanteric entry point to minimize the angle between the line of the proximal nail axis and the femoral long axis. Additionally, sufficiently deep central insertion of the lag screw was advised in both the sagittal and coronal planes.

## 1 Introduction

With the increase in the aging population, the incidence of intertrochanteric fractures is growing globally, and the direct costs associated with this condition are enormous (Adeyemi and Delhougne, 2019; Dong et al., 2023). Owing to the high 1-year mortality rates and other negative consequences, such as disability and depression, these fractures are commonly treated with operative management, which generally favors intramedullary nailing (Bhandari and Swiontkowski, 2017; Fischer et al., 2021; Liu P. et al., 2020). The remarkable benefits of surgical fixation are earlier mobility, accelerated recovery, and significant pain relief (Yu et al., 2015). Despite these advances, the failure of intramedullary nails is not rare and is estimated to be 2%–3% (Johnson et al., 2017). In previous literature, in patients with osteoporosis screw cut-out, cut-through, loss of reduction, or nonunion have been reported, which can result in various complications that may require reoperation (Ibrahim et al., 2019; Liu W. et al., 2020).

Compared with other conventional intramedullary nails, the InterTAN nail uniquely includes two lag screws in an integrated mechanism, which is designed to allow for linear compression and provide resistance to femoral head rotation for intertrochanteric fractures (Date et al., 2020). Additionally, the trapezoidal shape of the proximal end increases the contact surface on the lateral side of the implant for increased support and pressure resistance (Ruecker et al., 2009). Previous biomechanical studies in cadaveric models have provided evidence that the integrated dual-screw construct may provide significant stability and rotational resistance to the femoral head in older patients with osteopenia and unstable intertrochanteric fractures (Santoni et al., 2019; Santoni et al., 2016).

Although several design improvements to the InterTAN nail have been made, the importance of the correct placement of lag screws in the femoral head cannot be overemphasized. Earlier studies have concluded that the lag screws should be positioned within the central quarter of the femoral head, and an increased tip-apex distance was deemed unacceptable (Socci et al., 2017; Yam et al., 2017). Tip-apex distance was the sum of the distance in millimeters measured from the tip of the lag screw to the apex of the femoral head after controlling for magnification. If the screws are not placed optimally, the risk of ‘cut-out’ and ‘cut-through’ remains (Mayor et al., 2024; Socci et al., 2017). The purpose of this study was to investigate the reasons for an increased tip-apex distance of InterTAN nail lag screws, and how to avoid this phenomenon and reduce the risks of a cut-out, and finally establish a foundation for more effective treatment of intertrochanteric fractures in the elderly, which is crucial for enhancing their quality of life.

## 2 Methods

This retrospective study was conducted at our hospital between January 2017 and March 2022. Patients with intertrochanteric fractures, who underwent InterTAN nail insertion with some mobility before the fracture and had complete medical and radiological records, identified using the hospital information system were included in the study. The exclusion criteria included individuals under the age of 60 years, those with history of fracture or surgery on the contralateral hip, those with pathologic fractures, and those in whom the position of the lag screws could not be identified on radiographs.

**FIGURE 1:**
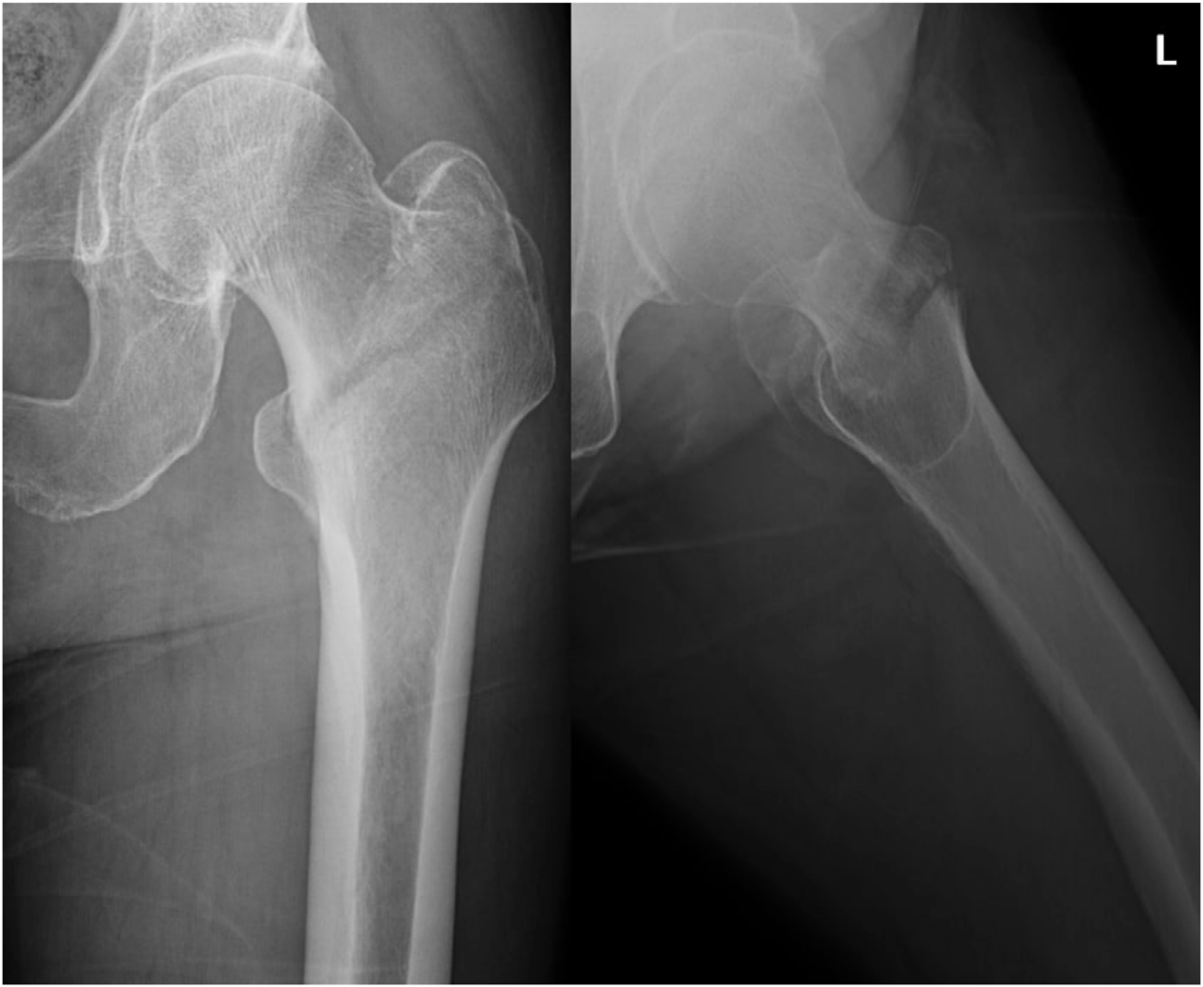
Preoperative radiographs.

Once a patient was extracted from our information system and included, demographic data, such as age and sex, were recorded. Preoperative radiographs (Figure 1) and computed tomography scans were recorded along with the measurement of AO foundation-Orthopedic Trauma Association (AO-OTA) classification (Marsh et al., 2007), integrity of the lateral wall and medial calcar, neck–shaft angle, and length of the femoral neck of the non-fractured side. Using radiographs obtained on the first postoperative day, the neck-shaft angle, tip-apex distance, placement of the lag screw within the femoral head, restoration of the lateral wall and medial calcar, length of the femoral neck, angle between the line of the proximal nail axis and the femoral long axis, and distance between the line of the proximal nail axis and the lateral trochanteric wall were assessed.

Based on the integrity and comminution, the lateral wall and medial calcar were defined as either intact (or two-part and without any translation or with less than one cortical thickness of the lateral or medial cortex), a simple disruption (two-part and more than one cortical thickness of the lateral or medial cortex) or comminuted (with ≥ three fragments).

The neck-shaft angle of the non-fractured side was measured between the line of the femoral neck axis and the femoral long axis (Boese et al., 2016). The vertical length of the femoral neck was measured using a line drawn perpendicular to the femoral long axis through the top of the lesser trochanter, and the distance between the center of the femoral head and the line was considered the vertical length (Marmor et al., 2012).

Lag screws were placed in both the sagittal and coronal planes. The sagittal plane options included center-center, center-inferior, center-superior, superior, and inferior, whereas the coronal plane options included center, posterior, and anterior.

**FIGURE 2:**
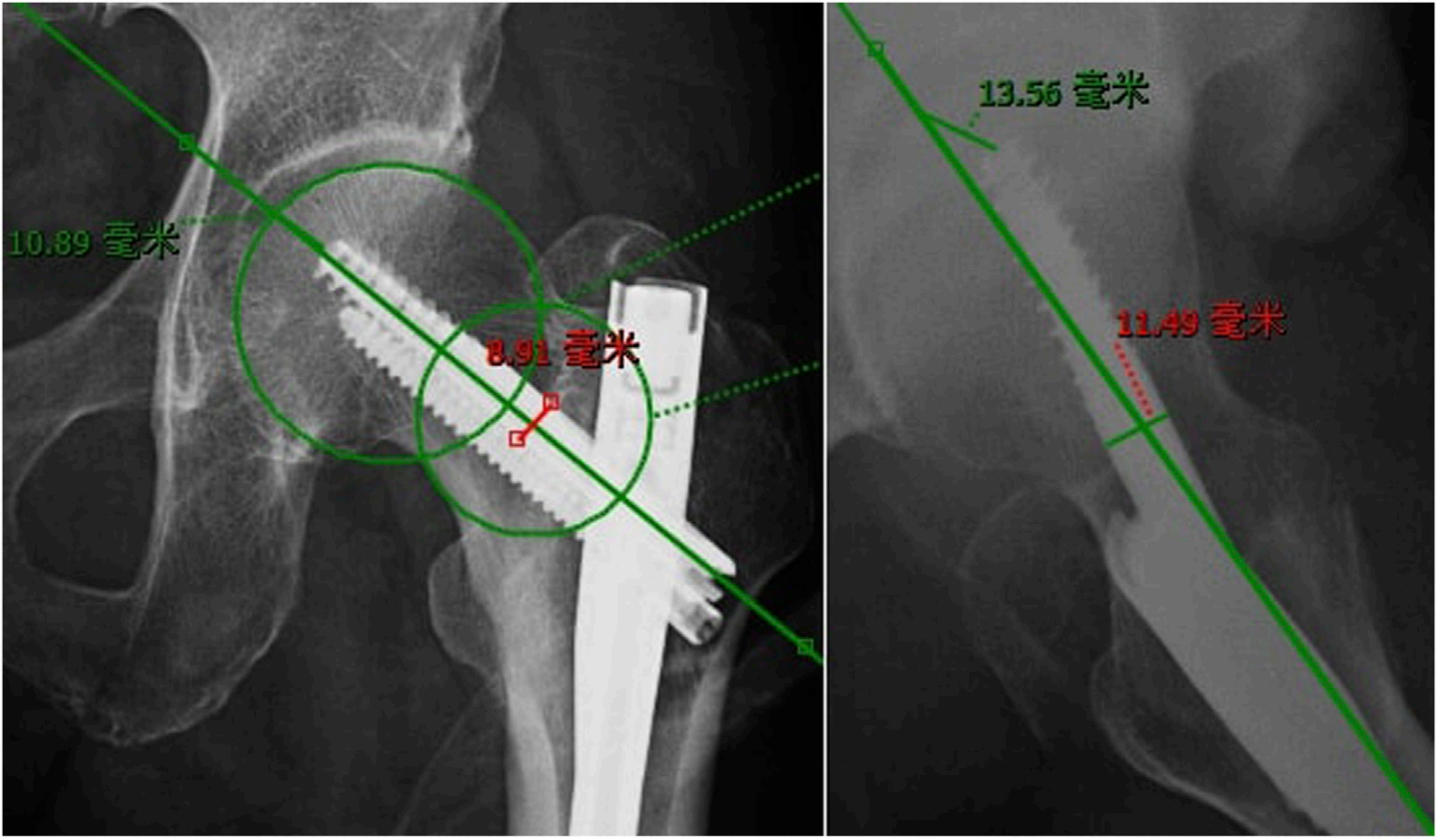
Measurement of the tip apex distance on the anteroposterior and lateral radiographs (mm).

The tip-apex distance (Figure 2), namely, the sum of the distance in millimeters measured from the tip of the lag screw to the apex of the femoral head after controlling for magnification, was calculated using anteroposterior and lateral radiographs (Baumgaertner et al., 1995). Many studies have identified that tip-apex distance was one of the most important factors of cut-out.

The reduction quality of the lateral wall, as previously described (Hao et al., 2019; Yoon et al., 2020) was defined as optimal reduction (no translation of the lateral cortex), acceptable reduction (translation of the lateral cortical continuity of less than one cortical thickness), or unacceptable reduction (translation of the lateral cortical continuity of more than one cortical thickness) on anteroposterior radiographs.

The reduction quality of the medial calcar was as previously described (Chang et al., 2015). Moreover, three types of medial cortex reductions including anatomical reduction, positive medial cortex support, and negative medial cortex support reduction were identified. The anatomic reduction was the complete cortex-to-cortex contact between the head-neck fragment and the femoral shaft. Positive or negative medial cortex support was defined as the displacement of the head-neck fragment medially or laterally to the superior medial edge of the shaft fragment. Anatomical and positive medial cortex support reductions were considered acceptable.

**FIGURE 3:**
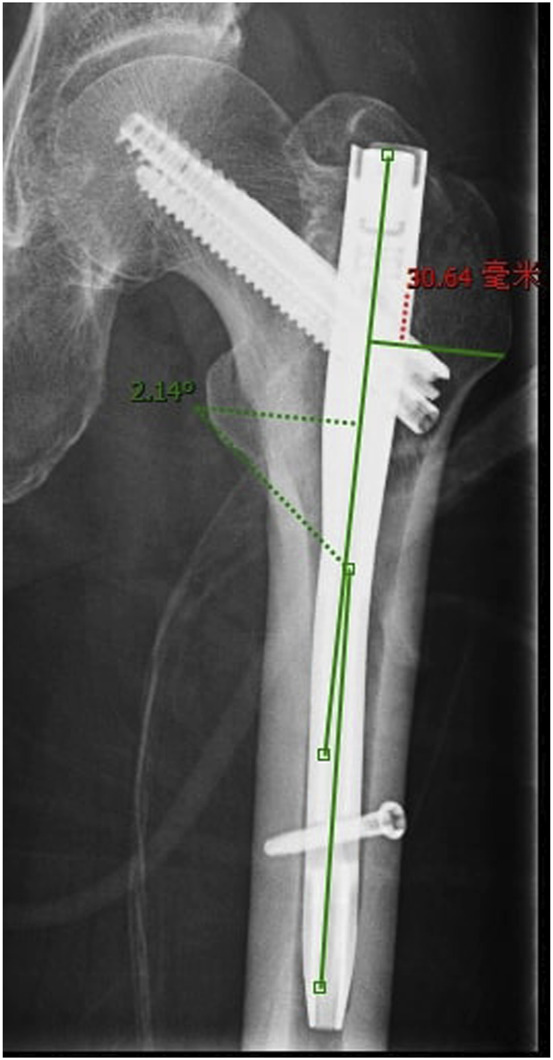
Measurement of the great trochanteric entry point, both the angle and distance.

The distance between the line of the proximal nail axis and the lateral trochanteric wall and the angle between the line of the proximal nail axis and the femoral long axis were measured to evaluate the greater trochanteric entry point of the intramedullary nail (Figure 3).

One reviewer completed the medical and radiological data collection and filled out the form. Another reviewer performed checks.

## 3 Statistical analysis

Multivariate logistic regression analysis was performed to evaluate the statistically significant variables of the tip-apex distance. By considering the variables individually, a backward likelihood ratio test was performed to carefully select the final best model. Statistical significance was set at *p* < 0.05, and all tests were two-sided. The R software (version 4.1.2) was used for all analyses.

## 4 Results

In total, 102 patients with intertrochanteric fractures were included in this study. The mean age of the patients was 81 years, with 28 men (27.5%) and 74 women (72.5%). Additionally, 47 (46.1%) patients had injuries on the left side, whereas the remaining 55 (53.9%) sustained injuries on the right side. The average tip-apex distance measured 22.4 ± 7.1 mm, ranging from 9.3 to 48.0 mm, and a mean angle of 2.9°between the line of the proximal nail axis and the femoral long axis was found.

Initially, after adjusting variables, multivariable logistic regression analyses demonstrated that comminuted lateral wall (beta −0.575; 95% confidence interval [CI] −1.02∼-0.12), optimal reduction (beta, 0.437; 95%CI, 0.030∼0.844), and unacceptable reduction of the lateral wall (beta, 0.572; 95%CI, 0.118∼1.02) were independent factors for tip-apex distance. However, these variables were removed because of multicollinearity.

Finally, the length of the femoral neck on the non-fractured side, placement of the lag screw in the sagittal plane (center-inferior, superior), the coronal plane (posterior), and the angle between the line of the proximal nail axis and the femoral long axis were statistically significant factors for the tip-apex distance (Table 1). Furthermore, the first three had a positive effect on the tip-apex distance, whereas the latter had a negative effect.

**TABLE 1:** Statistical results of the multivariate model.

Variable	Sample	Estimate (95% CI)	P-Value
AO classification			
31A1	24 (23.5%)	Reference	
31A2	69 (67.6%)	−0.08 (−0.212 to 0.047)	0.211
31A3	9 (8.8%)	0.110 (−0.098–0.320)	0.297
Length of the femoral neck of the non-fractured side	39.7 mm (28.4–57.9)	0.025 (0.014–0.035)	<0.0001
Placement of the lag screw in the sagittal plane			
Center-center	40 (39.2%)	Reference	
Center-inferior	18 (17.6%)	0.237 (0.088–0.387)	0.002
Center-superior	20 (19.6%)	0.025 (−0.122–0.173)	0.729
Superior	21 (20.6%)	0.259 (0.114–0.404)	0.0006
Inferior	3 (2.9%)	0.146 (−0.179–0.472)	0.374
Placement of the lag screw in the coronal plane			
Center	67 (65.7%)	Reference	
Posterior	15 (14.7%)	0.271 (0.116–0.425)	0.0007
Anterior	20 (19.6%)	0.136 (−0.005–0.279)	0.058
Distance between the line of the proximal nail axis and the lateral trochanteric wall	25.2 mm (6.5–35.0)	−0.011 (−0.025,0.0036)	0.14
Angle between the line of the proximal nail axis and the femoral long axis	2.9° (−2.4–6.0)	−0.038 (−0.736 to −0.002)	0.033

No statistically significant differences were observed in the AO-OTA classification or the distance between the line of the proximal nail axis and the lateral trochanteric wall.

## 5 Discussion

Intertrochanteric femoral fractures are associated with high morbidity and mortality rates. Various intramedullary systems and sliding hip screws have been used in the treatment of intertrochanteric femoral fractures, currently a trend exists towards increased use of intramedullary nail fixation (Grønhaug et al., 2022). Regardless of the implant chosen, poor patient outcomes such as varus collapse and cut-out continue to occur (Sisman et al., 2022). Comparison of the InterTAN nail with integrated compression screws and other available intramedullary nails in the treatment of intertrochanteric fractures displayed a reduced incidence of implant-related failure and re-intervention rates (Quartley et al., 2022). However, the risk of cut-out in unstable trochanteric fractures has been reported in recent studies (Liu P. et al., 2020). Many studies have identified that tip-apex distance, lag screw placement in the head, as well as neck-shaft angles, are important factors in predicting cut-out, with tip-apex distance being the most important factor (Oner et al., 2021; Yam et al., 2017). The tip-apex distance is also considered a radiographic index of implant placement in the head. Moreover, the importance of implant positioning has been recognized in the previous literature (Baumgaertner et al., 1995; Buyukdogan et al., 2017). This study aimed to evaluate the risk factors for high tip-apex distance after internal fixation with the InterTAN nail, which is not well studied to the best of our knowledge. We identified the placement of the lag screw in both the sagittal and coronal planes, the entry point of the InterTAN nail, and the length of the femoral neck on the non-fractured side as factors related to the issue.

Baumgaertner et al. (1995) established the concept of tip-apex distance to describe the position of a lag screw in the femoral head of a sliding hip screw device. The recommended tip-apex distance for this type of device is less than 25 mm, and this standard has been extrapolated to Cephalomedullary nail (Baumgaertner et al., 1995; Lee et al., 2022). Yam et al. (2017) studied 340 patients who underwent Proximal Femoral Nail Antirotation-II and identified that a tip-apex distance >27 mm increased the risk of the cut-out. Coviello et al. (2024) found a tip-apex distance value of 29.5 mm to be associated with a risk of cut-out in double-screw nails, when good fracture reduction is granted. Furthermore, Buyukdogan et al. (2017) study of 118 patients with hip fractures documented no reoperation when the tip-apex distance was less than <21.7 mm, and the overall mean tip-apex distance was 19.7 ± 4.8 mm. However, this value has not been extensively studied for InterTAN nails. As the InterTAN nail comprises two interlocking screws that form a worm gear, we measured the distance between the apex of the femoral head and the tip of the proximal lag screw on both anteroposterior and lateral radiographs. The overall mean tip-apex distance in our study was 22.4 mm, which is consistent with the results of previous studies.

The tip-apex distance was the sum of the distance from the tip of the lag screw to the apex of the femoral head on both anteroposterior and lateral radiographs after controlling for magnification (Baumgaertner et al., 1995). Therefore, the optimal tip-apex distance depends on the precise placement of the lag screw in both the sagittal and coronal planes. The optimal position of the lag screw in the femoral head has been suggested by numerous authors to be either center-center or center-inferior in the anteroposterior view, and centrally located in the lateral view (Konda et al., 2023; Lee et al., 2022). In this study, ideal lag screw placement was determined in 34% (35) of 102 cases, and the mean tip-apex distance was 20.3 mm. However, inadequate lag screw insertion depth can pose challenges in obtaining a suitable tip-apex distance in certain cases. Of the 35 cases, 7 had a tip-apex distance greater than 25 mm, with a maximum value of 35.7 mm. This may be the reason why the center-inferior placement of the lag screw in the sagittal plane found to be statistically significant factors for tip apex distance. 8 of the 35 cases were the center-inferior placement of the lag screw in the sagittal plane and the mean tip apex distance was 25.4 mm with a maximum value of 35.7 mm.

In the current study, we observed a significant correlation between placing the lag screw tip out of the center of the anteroposterior and lateral views and increased tip-apex distance. Other studies have also reported a connection between incorrect screw placement and an elevated risk of cut-out. Yam et al. (2017) reported a high risk of cut-out associated with posterior and superior screw positioning, whereas Şişman et al. (Sisman et al., 2022) discovered that localizations other than central or inferior on anteroposterior radiographs, as well as central on lateral radiographs, increased the risk of the cut-out. However, the aforementioned conclusions differ from those of a biomechanical study conducted by Oner et al. (2021) which identified that retroverted placement was the ideal lag screw placement for type A fractures in the sagittal plane. However, the ideal placement for type C fractures was anteverted. Although a debate is present in clinical and biomechanical studies on whether the lag screw placement should be centrally positioned on the lateral view and either centrally or inferiorly located on the anteroposterior view, it is commonly agreed that anterior placement of the lag screw on the lateral view poses an increased risk of screw cut-out (Konda et al., 2023). We identified that specifically posterior or anterior placement of the lag screws tended towards a high risk of increased tip-apex distance among cohorts in the >25 mm range.

Our study also demonstrated that the entry point of the InterTAN nail correlated with variations in the tip-apex distance. Additionally, positioning the implant correctly from the beginning to achieve sustained fracture reduction and accurate overall coronal and sagittal plane alignment of the implant is important (Lee et al., 2022). In the work done by Pan et al. (2017), they concluded that the medial posterior trochanteric entry point resulted in excellent nail and helical blade placement. In the surgical strategies recommended by Lee et al. (2022), the preferred method was to position the long axis of the implant in alignment with the femoral canal, approximately 5 mm posterior to the tip of the trochanter. In our study, we identified a mean angle of 2.9° between the line of the proximal nail axis and femoral long axis, and increasing this angle resulted in a smaller tip-apex distance.

Another finding of our study was that the length of the femoral neck on the non-fractured side was positively correlated with an increased tip-apex distance. This could also be attributed to the central insertion of the lag screw, which was not sufficiently deep in cases with a longer femoral neck.

This study has certain limitations. First, this was a retrospective study with no estimation of sample size. However, statistical principles consider a sample size of 100 to meet the most stringent rule of thumb (Norman et al., 2012), and we had 102 patients. Second, we used only one type of intramedullary nail: the InterTAN nail with angle-stable lag screws. No other types of intramedullary nails were included. In the biomechanical study by Wang C et al., they introduced a new implant and compared it with proximal femoral nail antirotation (PFNA) and InterTAN for the treatment of intertrochanteric fractures and concluded that this new implant had the best mechanical properties for the treatment of intertrochanteric fractures (Wang et al., 2024). Therefore, in future studies, we will compare factors that influence the increase in tip apex distance between more different intramedullary nails.

## 6 Conclusion

In conclusion, objective surgical correction using the InterTAN nail should focus on the proper positioning of the lag screw in both the sagittal and coronal planes. Moreover, utilizing a medial trochanteric entry point to minimize the angle between the proximal nail axis and the femoral long axis while ensuring sufficiently deep and central insertion of the lag screw is recommended. This approach facilitates a smaller tip apex distance and more effective treatment of intertrochanteric fractures in the elderly to enhance their quality of life and promote the rehabilitation.

## Data Availability

The raw data supporting the conclusions of this article will be made available by the authors, without undue reservation.
